# Efficacy and safety of intranasal midazolam versus intranasal ketamine as sedative premedication in pediatric patients: a meta-analysis of randomized controlled trials

**DOI:** 10.1186/s12871-022-01892-2

**Published:** 2022-12-22

**Authors:** Bingchen Lang, Huiqing Wang, Yuzhi Fu, Linan Zeng, Liang Huang, Qianqian Zhang, Shouming Chen, Zhi-jun Jia, Lingli Zhang

**Affiliations:** 1 Department of Pharmacy, West China Second University Hospital, Sichuan University, Chengdu, China; 2 Evidence-Based Pharmacy Center, West China Second University Hospital, Sichuan University, Chengdu, 610041 China; 3 Key Laboratory of Birth Defects and Related Diseases of Women and Children, Ministry of Education, Chengdu, China; 4 National Medical Products Administration (NMPA) Key Laboratory for Technical Research On Drug Products In Vitro and In Vivo Correlation, Chengdu, China; 5 Medical Simulation Centre, West China Second University Hospital, Sichuan University, Chengdu, China; 6 Department of Anesthesiology, Chengdu Women’s and Children’s Central Hospital, Chengdu, China; 7 Department of Anesthesiology, West China Second University Hospital, Sichuan University, Chengdu, China; 8 Department of Biopharmaceutics, West China School of Pharmacy, Sichuan University, Chengdu, China

**Keywords:** Midazolam, Ketamine, Intranasal, Sedation, Pediatrics

## Abstract

**Background:**

Intranasal midazolam and ketamine have been widely used as sedative premedication in children. It is difficult to determine which one yields better sedative effects for clinical practice. We conducted the present meta-analysis by summarizing the evidences to evaluate the efficacy and safety of intranasal midazolam versus intranasal ketamine as sedative premedication in pediatric patients.

**Methods:**

We searched PubMed, Embase, and Cochrane Library from inception to April 2022. All randomized controlled trials (RCTs) used intranasal midazolam and ketamine as sedatives in children were enrolled. The risk of bias in RCTs was assessed by Cochrane risk of bias tool. Condition of parental separation, anesthesia induction or facemask acceptance, sedation level, different hemodynamic parameters and adverse events were considered as the outcomes in our study.

**Results:**

A total of 16 studies with 1066 patients were enrolled. Compared with midazolam, administration of intranasal ketamine might be associated with severer changes in hemodynamics parameters including mean blood pressure (SMD = -0.53, with 95% CI [-0.93, -0.13]) and heart rate (HR) (SMD = -1.39, with 95% CI [-2.84, 0.06]). Meanwhile, administration of intranasal midazolam was associated with more satisfactory sedation level (61.76% vs 40.74%, RR = 1.53, with 95%CI [1.28, 1.83]), more rapid onset of sedation (SMD = -0.59, with 95%CI [-0.90, -0.28]) and more rapid recovery (SMD = -1.06, with 95%CI [-1.83, -0.28]). Current evidences also indicated that the differences of various adverse effects between two groups were not significant.

**Conclusions:**

Given that administration of midazolam via intranasal route provides more satisfactory sedative level with less fluctuation of hemodynamics parameters and more rapid onset and recovery, it might be considered as the preferred sedative premedication for pediatric patients compared to ketamine. However, the widespread evidences with low or moderate quality indicated that superiority of intranasal midazolam in pediatric sedation needs to be confirmed by more studies with high quality and large sample size in future.

**Trial registration:**

The protocol of present study was registered with PROSPERO (CRD42022321348).

**Supplementary Information:**

The online version contains supplementary material available at 10.1186/s12871-022-01892-2.

## Introduction

For pediatricians and anesthesiologists, relieving anxiety or stress in children before surgeries and procedures should be a recurring concern. A previous report alleged that up to 60–70% of children have experienced significant stress anxiety before surgeries [1]. Possible reasons for such behavioral problem in children include their concerns about physical discomfort during surgeries or clinical procedures and their concerns about the condition of being separated from parents [2]. And unfamiliar hospital environment and lack of understanding about surgeries or clinical conditions frequently frighten pediatric patients and exaggerate their unpleasant experience. It results in uncooperative physically resistance from children at the time of parental separation, mask application, or induction of anesthesia [3]. Therefore, it’s necessary to pay particular attention to treating preoperative anxiety in pediatric patients.

Sedative premedications, which has been found to be more effective than behavioral intervention [4, 5], can allay anxiety, decrease emotional discomforts, facilitate parental separation, and lead to an atraumatic induction of anesthesia. As a short-acting anxiolytic drug, midazolam provides fast sedation and has become one of the most frequently used preanaesthetic medication in pediatric patients [6], and it has been revealed repeatedly to be superior to the behavioral preparation programs [7] (e.g., the parental presence). Ketamine, an N-methyl D-aspartate (NMDA) receptor antagonist, also produces sedative effect without respiratory depression and it has been used as sedative premedication in children [8].

However, anatomical factors of children, especially small veins and excess subcutaneous fat, make visualization of veins difficult. It would be challenging to obtain reliable vascular access in pediatric patients [9]. Hence, intranasal administration, an alternative route for intravenous administration without risk of needle-stick injuries and high vascular access skill requirements, has been widely used in pediatric sedation to ensure a high level of compliance in children undergoing sedative premedication [10].

Recent studies indicated that both two mentioned-above pharmacological approaches have been widely used as intranasal sedatives in children [11, 12]. And a growing number of studies shifted focus to comparison between intranasal ketamine and intranasal midazolam in pediatric sedation. Gharde et al*.* [13] suggested that separation of children from their parents was more smooth in ketamine group compared to midazolam group. Meanwhile, Hosseini Jahromi et al*.* [14] and Milési et al. [15]*.* indicated that intranasal midazolam was more effective than intranasal ketamine in reducing preoperative pediatric anxiety and in rapidly achieving adequate sedation. It is difficult to determine which one yields better sedative effects for clinical practice. Therefore, the inconsistent conclusions from recent published studies prompt us to perform a meta-analysis by summarizing the evidences to evaluate the efficacy and safety of intranasal midazolam versus intranasal ketamine as sedative premedication in pediatric patients.

## Methods

### Protocol and registration

According to the recommendations in the Preferred Reporting Items for Systematic Reviews and Meta-Analyses (PRISMA) statement [16] and Cochrane Handbook, we performed the present meta-analysis. The protocol for this review was registered on International Prospective Register for Systematic Reviews (PROSPERO) (https://www.crd.york.ac.uk/prospero, CRD42022321348).

### Search strategy

Electronic databases including PubMed, Embase, and Cochrane Library were searched from inception to April 20, 2022 by two authors (BL and YF). And we also considered academic search engine Google Scholar as the additional information source. “Infant”, “child”, “adolescent”, “midazolam”, “nasal”, “intranasal” and “randomized controlled trial” were considered as our search terms (Appendix S1). Only human studies published in English or Chinese were considered in our present study.

### Eligibility criteria

#### Participants

The participants of present study were children (< 18 years old) who experienced various surgical or diagnostic procedures.

#### Intervention and comparison

Using midazolam and ketamine via intranasal route as sedative premedication were considered as intervention and comparison.

#### Outcome measures

It is generally agreed that ideal features of pediatric sedation included satisfactory separation from parents, induction of anesthesia or facemask compliance, stable hemodynamic status and limited adverse effects, thus, number of patients with satisfactory separation from parents, number of patients with satisfactory induction or mask acceptance, and number of patients with satisfactory sedation level were considered as co-primary outcomes in our present study. And the secondary outcomes were as follows: Onset of sedation, recovery time, hemodynamic status and various adverse effects between two groups.

#### Study design

Only randomized controlled trials (RCTs) were considered.

#### Exclusion criteria

Reviews, conference abstracts, cases, comments, preclinical studies, protocol, ongoing trials, studies not published in English or in Chinese, and studies with inappropriate comparisons or unrelated outcome measures were excluded.

### Data extraction, and assessment of the risk of bias

Literature screening and data extraction were performed by two independent authors (BL and YF), and then they crosschecked with each other. After deleting the duplicated items from different databases, the irrelevant records were excluded by scanning titles and abstracts. Then full texts of the remaining records were obtained and perused by us. The general characteristics of all enrolled studies which met the criteria were collected in Table 1. The risk of bias in RCTs was assessed by Cochrane risk of bias tool [17] including following aspects: random sequence generation (generation of the randomization sequence), allocation concealment, blinding of outcome assessment, incomplete outcome data, and selective reporting. All clinical researches could have classified as low, high, or unclear risk of bias across above-mentioned five domains. Any disagreement will be resolved by consulting a third investigator.

**Table 1:** The general characteristics of the enrolled studies

Study (Reference)	Year	Type of surgery /procedure	Patient age range & ASA status	Patients enrolled (Gender: F/M, n)	Intranasal Midazolam dose	Intranasal Ketamine dose	Scale used for sedation measurement	Outcomes
Richard A et al*.*[18]	1993	Dental extraction	17–62 mo, Not mentioned	20 (Not mentioned): 1. Midazolam group: 10 2. Ketamine group: 10	0.4 mg/kg	3 mg/kg	10-points scale	I, III, V(a)
Kazemi AP et al*.*[19]	2005	Elective surgery	2–5 yr, ASA I-II	90 (Not mentioned): 1. Midazolam group: 50 2. Ketamine group: 40	0.2 mg/kg	5 mg/kg	4-points scale	I
Gharde P et al*.*[13]	2006	Elective corrective surgical procedure	1–10 yr, Not mentioned	40 (15/25): 1. Midazolam group: 20 2. Ketamine group: 20	0.2 mg/kg	10 mg/kg	4-points scale	I
Gautam SN et al*.*[20]	2007	Elective surgery	1–6 yr, ASA I-II	50 (Not mentioned): 1. Midazolam group: 25 2. Ketamine group: 25	0.2 mg/kg	5 mg/kg	4-points scale	I
Hosseini Jahromi SA et al*.*[14]	2012	Elective surgery	2–8 yr, ASA I	60 (32/28): 1. Midazolam group: 30 2. Ketamine group: 30	0.2 mg/kg	3 mg/kg	6-points scale	I
Mostafa G et al*.*[21]	2013	Bone marrow biopsy and aspirate	2–8 yr, ASA II	64 (Not mentioned) 1. Midazolam group: 32 2. Ketamine group: 32	0.2 mg/kg	5 mg/kg	4-points scale	I, IV
Surendar MN et al*.*[22]	2014	Dental treatment	4–14 yr, ASA I	42 (Not mentioned) 1. Midazolam group: 21 2. Ketamine group: 21	0.2 mg/kg	5 mg/kg	5-points scale	I, II, III, IV
Narendra PL et al*.*[23]	2015	Various surgical procedures	1–10 yr, ASA II	100 (Not mentioned) 1. Midazolam group: 50 2. Ketamine group: 50	0.2 mg/kg	5 mg/kg	5-points scale	I, II, V(b,c)
Fei et al*.*[24]	2017	Surgery for pediatric tumors	1–3 yr, ASA I-II	60 (23/37): 1. Midazolam group: 30 2. Ketamine group: 30	0.2 mg/kg	5 mg/kg	4-points scale	I, III, IV
Akçay ME et al*.*[25]	2018	Ear nose throat surgical procedures	1–10 yr, ASA I-II	40 (14/26): 1. Midazolam group: 20 2. Ketamine group: 20	0.2 mg/kg	5 mg/kg	6-points scale	I, IV
Milesi C et al*.*[15]	2018	Non-emergent endotracheal intubation	24–36 weeks, Not mentioned	60 (25/35): 1. Midazolam group: 27 2. Ketamine group: 33	0.2 mg/kg	2 mg/kg	4-points scale comprises three domains	I, II, IV
Alp H et al*.*[26]	2019	Transthoracic echocardiography	9–36 mo, Not mentioned	139 (Not mentioned): 1. Midazolam group: 70 2. Ketamine group:69	0.2 mg/kg	4 mg/kg	4-points scale	I, IV, V(b,c)
Jafarnejad S et al*.*[27]	2020	Obtaining peripheral Intravenous (IV) line in emergency care	2–8 yr, ASA I-II	70 (35/35): 1. Midazolam group: 35 2. Ketamine group: 35	0.2 mg/kg	5 mg/kg	Observational Scale of Behavioral Distress-Revised (zero to 23.5)	I, IV
Khoshrang H et al*.*[28]	2021	Urologic elective surgeries	2–6 yr, ASA I-II	71 (Not mentioned): 1. Midazolam group: 35 2. Ketamine group: 36	0.5 mg/kg	5 mg/kg	5-points scale	I, III, V(b,c)
Verma I et al*.*[29]	2021	Elective cardiac surgery	1–12 yr, ASA II- III	60 (23/37): 1. Midazolam group: 30 2. Ketamine group: 30	0.2 mg/kg	5 mg/kg	5-points scale	I, IV, V(b)
Abusinna RG et al*.*[30]	2022	minor elective surgical procedures	2–9 yr, ASA I-II	100 (52/48): 1. Midazolam group: 50 2. Ketamine group: 50	0.2 mg/kg	2 mg/kg	6-points scale	I, II, IV

I—Sedative effects of premedication (e.g. number of patients with satisfactory separation from parents, number of patients with satisfactory induction or mask acceptance, and number of patients with satisfactory sedation level)

II—Onset of sedation

III—Recovery time

IV—Hemodynamic status

V— Adverse effects (a. Respiratory depression and oxygen desaturation; b. Nauseas and vomiting; c. Agitation)

### Grading the quality of evidence

We used the Grading of Recommendations Assessment, Development, and Evaluation (GRADE) methodology [31] to assess the quality of evidence and strength of recommendations considering risk of bias, inconsistency, indirectness, imprecision, and publication bias. The quality of evidence was classified as high, moderate, low, or very low. The analysis was performed by using the GRADE profiler software (version 3.6, provided by the Cochrane collaboration).

### Statistical analysis

We used Review Manager software (Version 5.3.3, the Cochrane Collaboration 2014, the Nordic Cochrane Centre) for statistical analysis. Standardized mean difference (SMD) with 95% confidence interval (CI) was applied to estimate continuous variables, and risk ratio (RR) with 95% confidence interval (CI) and the Mantel–Haenszel method (fixed or random models) were used to analyze dichotomous data. And Heterogeneity was assessed through the I-squared (*I*^2^) test [32]. If significant heterogeneity (present at *I*^2^ > 50%) existed, the sensitivity analysis was considered by omitting each study separately, and the random effects model was applied; otherwise, the fixed-effects model would be considered. If sufficient studies (the number exceeds 10) were included for the primary or second outcomes [33], a funnel plot to explore the possibility of publication bias would be performed by us.

## Results

### Literature search results

**Fig. 1:**
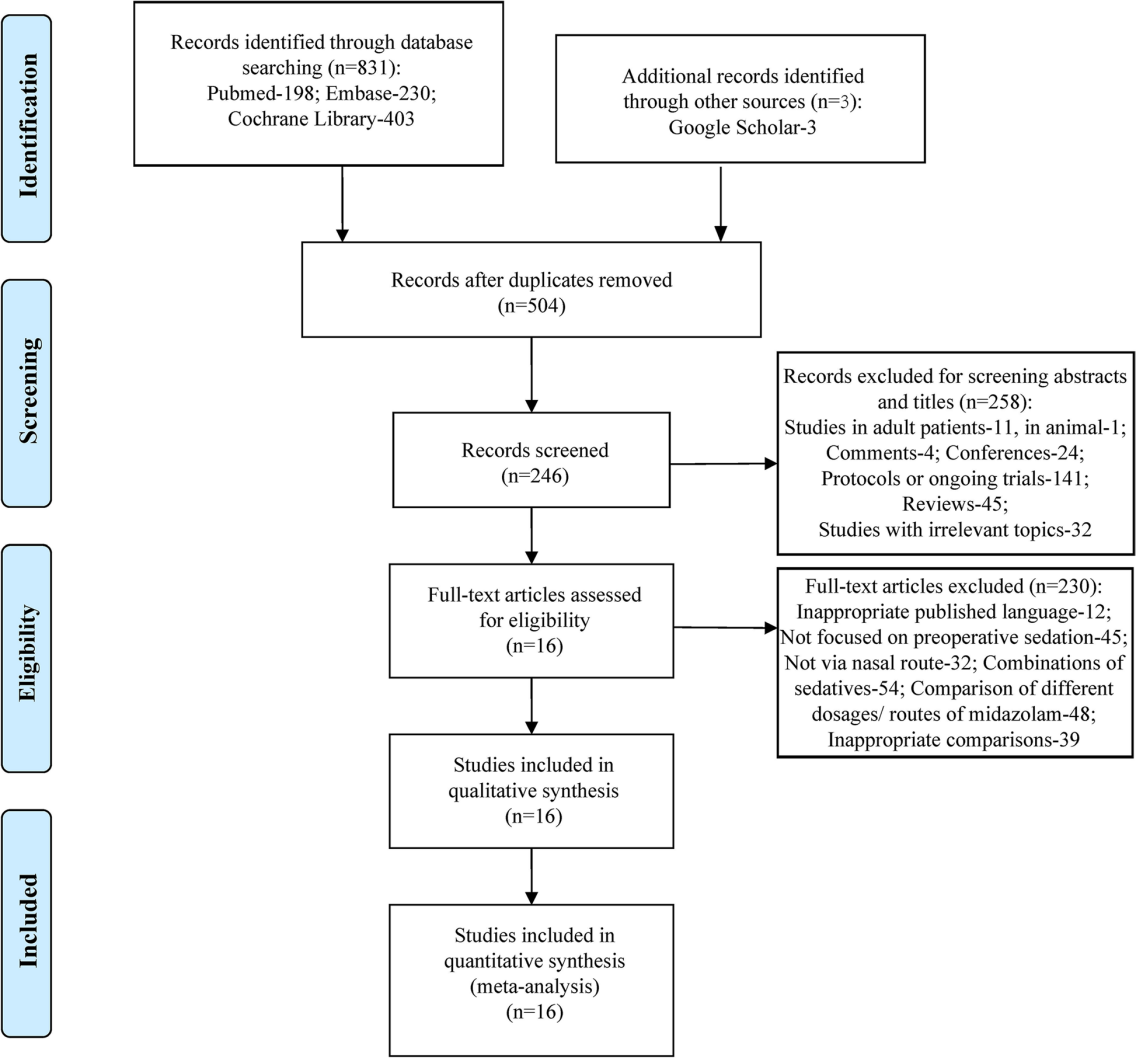
Flow chart of literature screening and the selection process

A total of 834 studies were identified initially after databases screening and additional source searching. And then we removed 330 duplicate records, and excluded 258 records by reviewing titles and abstracts. In these 258 excluded items, 11 were studies about adult patients, 1 was the study performed in animal, 4 were comments notes, 24 were conference news or abstracts, 141 were protocols or ongoing trials, 45 were reviews, and 32 were studies focused on irrelevant topics. And consequently 230 items were excluded by full-text reviewing, 12 studies were excluded based on language (4 were written in Spanish, 3 in German, 2 in French, 1 in Italian, 1 in Turkish, and 1 in Korean), 45 were studies not focused on preoperative sedation, 32 studies were concerned with sedatives via other routes of administration, 54 studies were concerned with combined medication, 48 were studies focused on comparison of different dosages and different routes of midazolam, and 39 studies were excluded owing to the inappropriate comparisons. Eventually, 16 studies were chosen in consequent analysis [13–15, 18–30]. The details of literatures identification are described in PRISMA flowchart (Fig. 1).

### Basic characteristics of enrolled studies

The enrolled studies were published from 1993 to 2022, and 1066 eligible pediatric patients (ages ranged from 24 weeks to 14 years) were involved in total. Intranasal midazolam at a dosage range of 0.2 mg/kg-0.5 mg/kg and intranasal ketamine at a dosage range of 2 mg/kg-10 mg/kg was given in children undergoing various types of surgery or procedure including cardiac surgeries, cancer surgeries, urologic surgeries, ear nose throat surgeries, corrective surgeries, dental treatment, bone marrow biopsy, endotracheal intubation, echocardiography and other elective surgeries. All studies in present analysis described the primary outcomes “number of patients with satisfactory sedation level, number of patients with satisfactory separation from parents, or number of patients with satisfactory induction or mask acceptance” [13–15, 18–30]. The outcome “Onset of sedation” was reported in 4 studies [15, 22, 23, 30], the outcome “Recovery time” was mentioned in 4 studies [18, 22, 24, 28], and 5 studies concerned the occurrence of different adverse effects [18, 23, 26, 28, 29]. The main characteristics of enrolled studies were summarized in Table 1.

### Risk of bias assessment

**Fig. 2:**
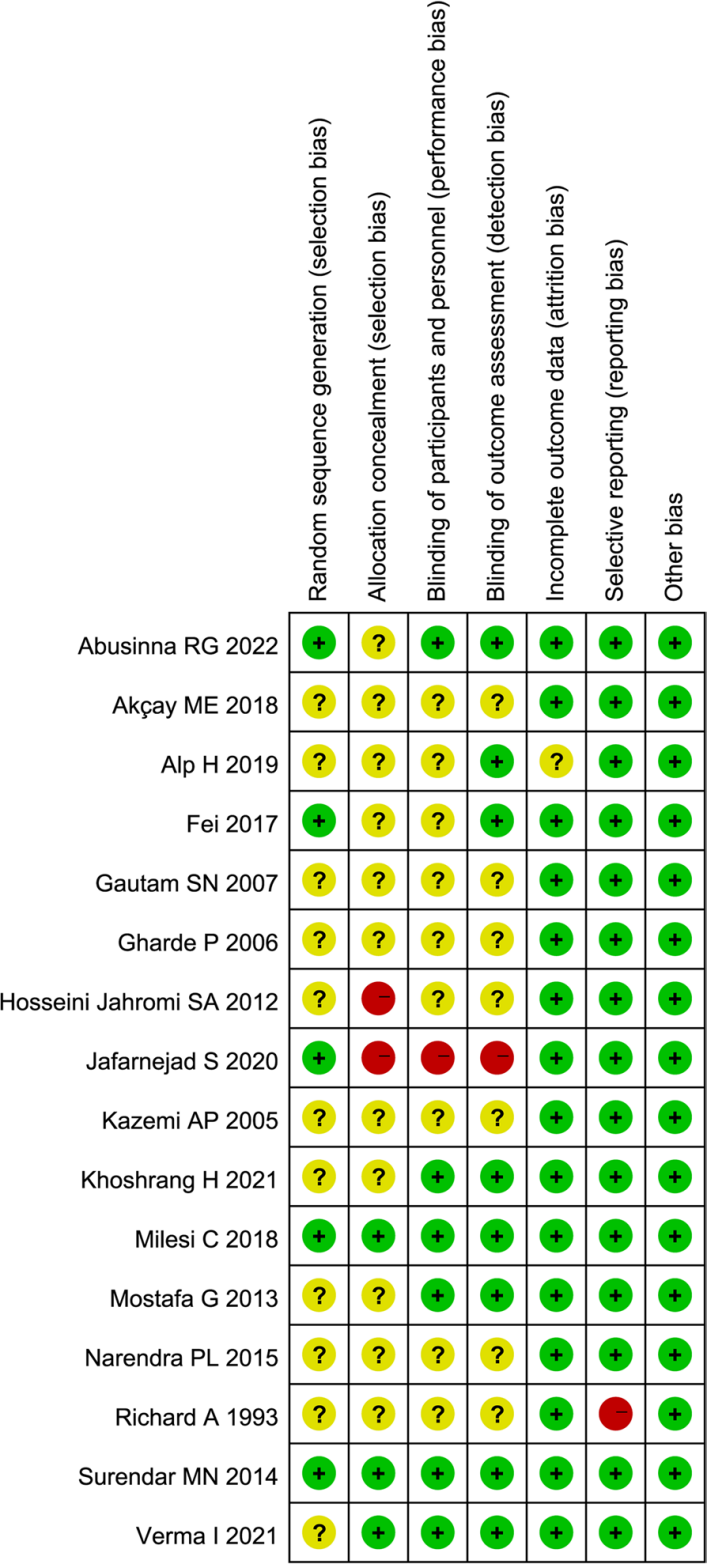
Risk of bias assessment of included studies. Green + dot, low risk of bias; yellow? dot, unclear risk of bias; red—dot, high risk of bias

Cochrane Collaboration’s risk of bias tool was used in evaluating validity and quality of included RCTs by us. In totally, 31.25% (5/16) studies described appropriate method of random sequence generation, only 18.75% (3/16) studies reported the allocation concealment, 37.50% (6/16) studies had low risk in blinding of participants and personnel domain, half of studies (8/16) described blinding procedure of outcome assessment. The detailed information about risk of bias assessment was showed in Fig. 2.

### Primary outcomes

#### Number of patients with satisfactory separation from parents

**Fig. 3:**
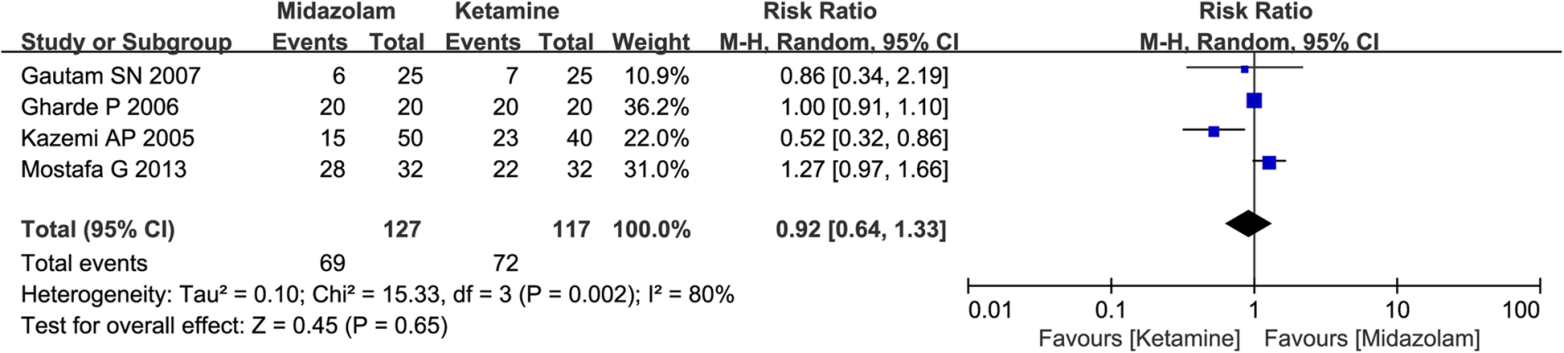
Forest plot depicting the meta-analysis for the outcome “Number of patients with satisfactory separation from parents” for intranasal midazolam versus intranasal ketamine

Four studies involving 244 pediatric patients described the number of patients with satisfactory separation from parents, and all of them focused attention on comparison between midazolam and dexmedetomidine. The random-effects model was chosen due to the existence of statistical heterogeneity. Results indicated that no significant differences were observed between midazolam group and ketamine group (54.33% vs 61.54%, RR = 0.92, with 95%CI [0.64, 1.33], P = 0.65, *I*^2^ = 80%; Fig. 3). According to GRADE summary of findings table, the quality of evidence for this outcome was very low. It was resulted from inconsistency (*I*^2^ > 50%) and imprecision (lack of events number) (Table S1).

#### Number of patients with satisfactory induction or mask acceptance

**Fig. 4:**
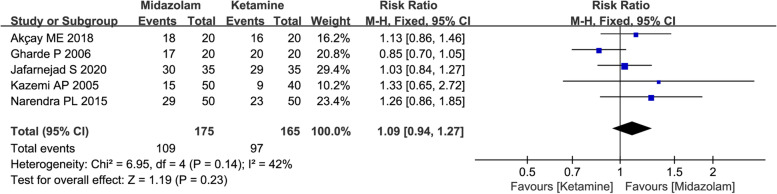
Forest plot depicting the meta-analysis for the outcome “Number of patients with satisfactory induction or mask acceptance” for intranasal midazolam versus intranasal ketamine

A total of 340 children in five studies reported the number of patients with satisfactory induction or mask acceptance. Given that limited statistical heterogeneity was detected among the study results (*I*^2^ = 42%), the fixed-effects model was used. The results of analysis also indicated that no significant differences were observed between two groups (62.29% vs 58.79%, RR = 1.09, with 95%CI [0.94, 1.27], P = 0.23, *I*^2^ = 42%; Fig. 4). As demonstrated as GRADE summary of findings table, the quality of evidence for this outcome was moderate, and imprecision (limited number of events) was considered as the main factor (Table S1).

#### Number of patients with satisfactory sedation level

**Fig. 5:**
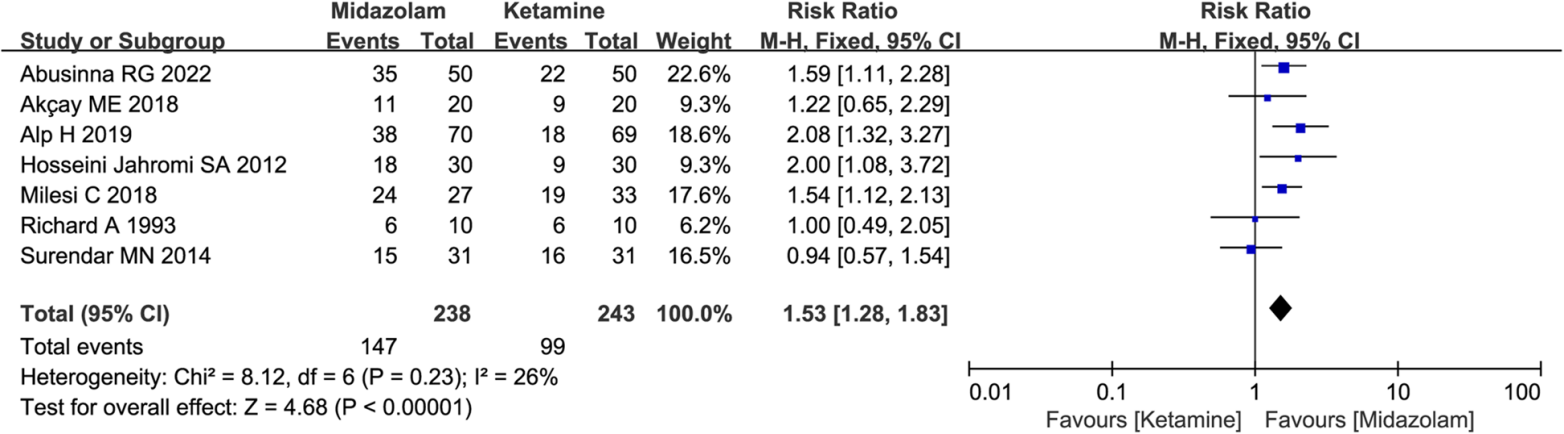
Forest plot depicting the meta-analysis for the outcome “Number of patients with satisfactory sedation level” for intranasal midazolam versus intranasal ketamine

Although the evaluation scales or scores about pediatric sedative level vary, according to review of Cravero et al*.*, [34], any sedation treatment that allows a procedure, for example, facilitating smooth anaesthetic induction, to be completed should be considered as the successful sedation. And in most of included literatures, cooperative behavior with minor fussing and struggle was served as an adequate sedation in pediatric patients. Seven studies including 472 pediatric patients were considered in analysis. Owing to absence of statistical heterogeneity (*I*^2^ = 26%), the fixed-effects model was chosen. The result indicated that the using of midazolam via intranasal route was associated with more satisfactory sedation level compared to intranasal ketamine (61.76% vs 40.74%, RR = 1.53, with 95%CI [1.28, 1.83], P < 0.0001, *I*^2^ = 26%; Fig. 5). The GRADE summary of findings table indicated that quality of evidence for present outcome was moderate. Imprecision (limited number of events) and high risk of bias were main factors (Table S1).

### Secondary outcomes

Results of secondary outcomes including hemodynamic parameters, onset of sedation, recovery time and various adverse effects were summarized in Table 2. Hemodynamic parameters including heart rate (HR), systolic blood pressure (SBP), mean blood pressure (MBP), and oxygen saturation were reported separately in 4 studies [21, 24–26], 1 study [21], 2 studies [24, 25], and 2 studies [21, 24]. The results indicated that intranasal ketamine was associated with significant higher value of MBP (SMD = -0.53, with 95% CI [-0.93, -0.13], *P* = 0.009; *I*^2^ = 0%) and oxygen saturation (SMD = -0.57, with 95% CI [-1.13, -0.02], *P* = 0.04; *I*^2^ = 57%). Additionally, the result indicated that intranasal ketamine might be associated with higher value of HR (SMD = -1.39, with 95% CI [-2.84, 0.06], *P* = 0.06; *I*^2^ = 96%). Meanwhile the results also indicated that intranasal midazolam was associated with more rapid onset of action (SMD = -0.59, with 95% CI [-0.90, -0.28], *P* = 0.0002; *I*^2^ = 0%) and more rapid recovery (SMD = -1.06, with 95% CI -1.06 [-1.83, -0.28], *P* = 0.008; *I*^2^ = 82%) compared to ketamine. The current evidences also indicated that the differences of adverse effects (*e.g.* agitation, oxygen saturation below 90%, nauseas and vomiting) between two groups were not significant.

**Table 2:** Secondary outcomes

Secondary outcomes	Number of studies (Reference no.)	Patients in Midazolam group (Incidence, %)	Patients in Ketamine group (Incidence, %)	*I*^2^ (%)	Risk ratio with [95% CI]	*P* value
Nauseas and vomiting	4 [23, 26, 28, 29]	16/185 (8.65%)	23/185 (12.43%)	66	0.89 [0.21, 3.69]	0.87
Agitation	3 [26, 28, 29]	16/155 (10.32%)	11/155 (7.10%)	0	1.45 [0.71, 2.94]	0.30
Oxygen saturation below 90%	1 [18]	0/10 (0.00%)	2/10 (20.00%)	-	-	-
Secondary outcomes	Number of studies (Reference no.)	Number of patients in Midazolam group	Number of patients in Ketamine group	*I*^2^ (%)	Standardized Mean difference with [95% CI]	*P* value
Onset of sedation	3 [15, 21, 22]	80	86	0	-0.59 [-0.90, -0.28]	0.0002*
Recovery time	3 [22, 24, 28]	86	87	82	-1.06 [-1.83, -0.28]	0.008*
Heart rate (HR)	4 [21, 24–26]	152	151	96	-1.39 [-2.84, 0.06]	0.06
Systolic blood pressure (SBP)	1 [21]	32	32	-	-	**-**
Mean blood pressure (MBP)	2 [24, 25]	50	50	0	-0.53 [-0.93, -0.13]	0.009*
Oxygen saturation	2 [21, 24]	62	62	57	-0.57 [-1.13, -0.02]	0.04*

### Sensitivity analysis and assessment of publication bias

According to the results, substantial heterogeneity only existed in analysis for one primary outcome “ Number of patients with satisfactory separation from parents” (*I*^*2*^ = 80%), however, the source could not be attributed to one particular study by sensitivity analysis; therefore, we applied random effects model in analysis. Given that each outcome included fewer than 10 studies, there were insufficient data for any publication bias analysis [33, 35].

## Discussion

Both midazolam and ketamine have been widely used in pediatric sedation. As an ultra-short acting sedative and anxiolytic, application of midazolam is frequently associated with rapid onset and with better recovery profile [36]. And ketamine is also one sedative option for its hypnotic and analgesic effect [37]. Compared with intravenous administration, intranasal administration is noninvasive and is highly preferred for pediatric sedation. It provides rapid drug absorption and leads to high drug bioavailability. According to studies published in recent years, two sedatives have been regarded as the most commonly used preoperative sedatives via intranasal route. However, the inconsistent conclusions from recent published studies [13–15] indicated that it is difficult to determine the preferred one for clinical sedation. To our knowledge, no relevant study has been established to examine the effects between two medications via intranasal route in pediatric sedation. Therefore, we performed present meta-analysis to evaluate efficacy and safety of two interventions as sedative premedication in pediatric patients.

The main objectives of preoperative sedation and optimal sedative level in children may vary with the specific procedure, but generally encompass alleviating anxiety, controlling excessive movement and facilitating parental separation. Therefore, parental separation, anesthesia induction or facemask acceptance, sedation level were considered as the major concerns in present study. In our study, a total of 16 RCTs including over 1000 pediatric patients were included. The current results of primary outcome indicated that intranasal premedication of midazolam might provide more satisfactory sedation level compared to ketamine (61.76% vs 40.74%, RR = 1.53, with 95%CI [1.28, 1.83], *P* < 0.0001, *I*^2^ = 26%; Fig. 5). However, the results also indicated that no significant differences were observed between two groups in number of patients with satisfactory separation from parents and in number of patients with satisfactory induction or mask acceptance. The inconsistent results from these co-primary outcomes might be resulted from small numbers of studies included in analysis, especially for the first two outcomes (Fig. 3 and Fig. 4), and the limited number of events was also the contributing factor to imprecision and unreliability.

Several studies suggested that intranasal midazolam should be considered as one safe medication for its minor influence on respiratory and cardiovascular parameters, [23, 38]. In our study, several side effects including nauseas/vomiting, agitation, and several common hemodynamics parameters were evaluated. The results of secondary outcomes indicated that children received ketamine via intranasal route was associated with higher value of hemodynamics parameters compared to midazolam. In fact, acute changes, especially the increased blood pressure and heart rate, in the cardiovascular status of patients are always considered as the side effects of ketamine, which were predominantly attributed to its sympathomimetic actions by direct stimulation of central nervous system structures [39]. And actually, most cardiovascular effects were reported as occurring during or immediately after intravenous ketamine administration [40]. According to traditional view, nauseas/vomiting and agitation may be resulted mainly from the perioperative use of inhalational anaesthesia and opioids [41, 42]. Although views differ widely on whether these premedications are effective in alleviating the side effects [43–45], current evidences from present study demonstrated that no difference was found in incidences of agitation, nauseas and vomiting between two groups. And our study also indicated that children received intranasal midazolam as premedication might be associated with rapid onset of action and recovery profile, which strengthened the findings from several previous studies [46, 47].

There are some limitations in our present study should be noted. One would be widespread low or moderate quality in outcomes evaluated by GRADE system. Inconsistency (high heterogeneity) and imprecision (lack of events number) might be considered as main factors. Another limitation was the lack of studies with large sample size in most outcomes of our meta-analysis. In present study, some unpublished materials (e.g., data from some registered ongoing trials) and articles published in languages other than English or Chinese were not included as they did not provide sufficient accessible information to allow our analysis. To compensate for the lack of information resource, we performed a thorough search for grey literature from websites “http://www.greylit.org/” and “http://greyguide.isti.cnr.it/” by using terms “midazolam” and “ketamine” (Accessed 19, Oct, 2022), but no results were found. Moreover, a search strategy as comprehensive as possible and a search considered additional source from Google scholar were also applied by us. However, the number of enrolled pediatric patients was still insufficient, studies with large sample size in future were required to draw more reliable conclusions. In addition, owing to each outcome in present study included fewer than 10 studies, data for publication bias analysis were insufficient and the analysis did not conducted by us [33, 35].

Moreover, considering that sedating children for diagnostic or surgical procedures has evolved into an important clinical issue involving diverse specialties outside of anesthesia. The emphasis in future should be placed on evaluation the optimal sedative premedication option with optimal dose range in different procedures.

## Conclusions

Both intranasal midazolam and intranasal ketamine have been widely used in pediatric sedation for many years. Based on all current evidences gathered from our analysis, no significant differences are found in adverse effects (e.g. agitation, oxygen saturation below 90%, nauseas and vomiting) between two groups., but intranasal midazolam provides more adequate sedative level, more rapid onset and recovery with less fluctuation of hemodynamics parameters, therefore, it might be considered as the preferred intranasal sedative option for pediatric patients compared to ketamine. However, overall low and moderate quality evidences in primary outcomes evaluated by GRADE system suggest that superiority of intranasal midazolam in pediatric sedation needs to be validated, and more studies with high quality and large sample size in future will be needed to draw a more reliable conclusion.

## Supplementary Information

**Additional file 1:** **Appendix S1.** Search Strategy.

**Additional file 2: Appendix S2.** Prisma checklist.

**Additional file 3:** **Table S1.** GRADE summary of findings table.

## Data Availability

All data generated or analyzed during this study are included in this published article [and its supplementary information files].

## Abbreviations

RCTs: Randomized controlled trials
SMD: Standardized mean difference
CI: Confidence interval
RR: Risk ratio
HR: Heart rate
SBP: Systolic blood pressure
MBP: Mean blood pressure
GRADE: Grading of recommendations assessment, development, and evaluation
PRISMA: Preferred reporting items for systematic reviews and meta-analyses statement
