# Supplementary material for: Efficacy and safety of intranasal midazolam versus intranasal ketamine as sedative premedication in pediatric patients: a meta-analysis of randomized controlled trials
Source: BMC Anesthesiol. 2022 Dec 22;22:399. doi: 10.1186/s12871-022-01892-2 (PMC9773574; doi:10.1186/s12871-022-01892-2)
Supplement: Supplementary file 1 — Additional file 1: Appendix S1. Search Strategy. [file 12871_2022_1892_MOESM1_ESM.docx]

**Pubmed**

| **Search** | **Query** |
| --- | --- |
| #1 | Infant [mesh] |
| #2 | newborn* [tiab] or neonat* [tiab] or infant* [tiab] or infancy [tiab] or baby [tiab] or babies [tiab] or toddler* [tiab] |
| #3 | #1 OR #2 |
| #4 | Child [mesh] |
| #5 | Pediatrics [mesh] |
| #6 | p?ediatric* [tiab] or child* [tiab] or kindergar* [tiab] or preschool* [tiab] or kid [tiab] or kids [tiab] or schoolchild* [tiab] or “school age” [tiab] or schoolage [tiab] or preteen* [tiab] or youth* [tiab] or prepubescent* [tiab] |
| #7 | #4 OR #5 OR #6 |
| #8 | Adolescent [mesh] |
| #9 | adolesc* [tiab] or teen* [tiab] or youth* [tiab] or underage* [tiab] or “under age*” [tiab] or minor* [tiab] or juvenile* [tiab] or pubert* [tiab] or pubescen* [tiab] or “young people*” [tiab] or “young person*” [tiab] or “young adult*” [tiab] |
| #10 | #8 OR #9 |
| #11 | #3 OR #7 OR #10 |
| #12 | midazolam [tiab] OR midazolamum [tiab] OR buccolam [tiab] OR seizalam [tiab] OR Anquil [tiab] OR Benzosed [tiab] OR Dalam [tiab] OR Damizol [tiab] OR Demizolam [tiab] OR Doricum [tiab] OR Dormicum [tiab] OR Dormid [tiab] OR Dormipron [tiab] OR Dormire [tiab] OR Dormitol [tiab] OR Dormixal [tiab] OR Dormonid [tiab] OR Drimnorth [tiab] OR Epistatus [tiab] OR Flormida [tiab] OR Fulsed [tiab] OR Garen [tiab] OR Gobbizolam [tiab] OR Hipnazolam [tiab] OR Hipnoz [tiab] OR Hypnofast [tiab] OR Hypnovel [tiab] OR Ipnovel [tiab] OR Nocturna [tiab] OR Setam [tiab] OR Talentum [tiab] OR Terap [tiab] OR Versed [tiab] |
| #13 | nasal* OR intranasal* OR "nasal administration" OR "nasal drug administration" OR "nasal instillation" OR "nasal medication" |
| #14 | (randomized controlled trial [pt] OR controlled clinical trial [pt] OR randomized [tiab] OR placebo [tiab] OR clinical trials as topic [mesh: noexp] OR randomly [tiab] OR trial [ti]) NOT (animals [mh] NOT humans [mh]) |
| #15 | #11 AND #12 AND #13 AND #14 |
| Items:198 | |

**Embase**

| **Search** | **Query** |
| --- | --- |
| #1 | 'Infant'/exp |
| #2 | (newborn* or neonat* or infant* or infancy or baby or babies or toddler*):ab,ti |
| #3 | #1 OR #2 |
| #4 | 'child'/exp |
| #5 | 'pediatrics'/exp |
| #6 | (paediatric*or pediatric* or child* or kindergar* or preschool* or kid or kids or schoolchild* or 'school age' or schoolage or preteen* or youth* or prepubescent*):ab,ti |
| #7 | #4 OR #5 OR #6 |
| #8 | 'adolescent'/exp |
| #9 | (adolesc* or teen* or youth* or underage* or "under age*" or minor* or juvenile* or pubert* or pubescen* or "young people*" or "young person*" or "young adult*"):ab,ti |
| #10 | #8 or #9 |
| #11 | (#3 or #7 or #10) and [embase]/lim |
| #12 | (midazolam or midazolamum or buccolam or seizalam or Anquil or Benzosed or Dalam or Damizol or Demizolam or Doricum or Dormicum or Dormid or Dormipron or Dormire or Dormitol or Dormixal or Dormonid or Drimnorth or Epistatus or Flormida or Fulsed or Garen or Gobbizolam or Hipnazolam or Hipnoz or Hypnofast or Hypnovel or Ipnovel or Nocturna or Setam or Talentum or Terap or Versed):ab,ti |
| #13 | #12 and [embase]/lim |
| #14 | nasal* or intranasal* or "nasal administration" or "nasal drug administration" or "nasal instillation" or "nasal medication" |
| #15 | #14 and [embase]/lim |
| #16 | 'randomized controlled trial'/exp |
| #17 | 'controlled clinical trial'/exp |
| #18 | 'randomization'/exp |
| #19 | 'double blind procedure'/exp |
| #20 | 'single blind procedure'/exp |
| #21 | random*:ab |
| #22 | trial*:ab |
| #23 | #17 OR #18 OR #19 OR #20 OR #21 OR #22 |
| #24 | 'human'/exp |
| #25 | #23 AND #24 AND [embase]/lim |
| #26 | #11 AND #13 AND #15 AND #25 |
| Items: 230 | |

**Cochrane Library**

| **Search** | **Query** |
| --- | --- |
| #1 | MeSH descriptor: [Infant] explode all trees |
| #2 | (newborn* or neonat* or infant* or infancy or baby or babies or toddler*):ti,ab,kw |
| #3 | #1 or #2 |
| #4 | MeSH descriptor: [Child] explode all trees |
| #5 | MeSH descriptor: [Pediatrics] explode all trees |
| #6 | (paediatric*or pediatric* or child* or kindergar* or preschool* or kid or kids or schoolchild* or 'school age' or schoolage or preteen* or youth* or prepubescent*):ti,ab,kw |
| #7 | #4 or #5 or #6 |
| #8 | MeSH descriptor: [Adolescent] explode all trees |
| #9 | (adolesc* or teen* or youth* or underage* or "under age*" or minor* or juvenile* or pubert* or pubescen* or "young people*" or "young person*" or "young adult*"):ti,ab,kw |
| #10 | #8 or #9 |
| #11 | #3 or #7 or #10 |
| #12 | (midazolam or midazolamum or buccolam or seizalam or Anquil or Benzosed or Dalam or Damizol or Demizolam or Doricum or Dormicum or Dormid or Dormipron or Dormire or Dormitol or Dormixal or Dormonid or Drimnorth or Epistatus or Flormida or Fulsed or Garen or Gobbizolam or Hipnazolam or Hipnoz or Hypnofast or Hypnovel or Ipnovel or Nocturna or Setam or Talentum or Terap or Versed):ti,ab,kw |
| #13 | (nasal* or intranasal* or "nasal administration" or "nasal drug administration" or "nasal instillation" or "nasal medication"):ti,ab,kw |
| #14 | #11 and #12 and #13 |
| Items:467 (64 reviews, 403 trials) | |
