# Supplementary material for: Efficacy and safety of intranasal midazolam versus intranasal ketamine as sedative premedication in pediatric patients: a meta-analysis of randomized controlled trials
Source: BMC Anesthesiol. 2022 Dec 22;22:399. doi: 10.1186/s12871-022-01892-2 (PMC9773574; doi:10.1186/s12871-022-01892-2)
Supplement: Supplementary file 3 — Additional file 3: Table S1. GRADE summary of findings table. [file 12871_2022_1892_MOESM3_ESM.docx]

**Table S1. GRADE summary of findings table**

| **Quality assessment** | | | | | | | **Summary of Findings** | | | | |
| --- | --- | --- | --- | --- | --- | --- | --- | --- | --- | --- | --- |
| **Participants (studies)**  **Follow up** | **Risk of bias** | **Inconsistency** | **Indirectness** | **Imprecision** | **Publication bias** | **Overall quality of evidence** | **Study event rates (%)** | | **Relative effect** (95% CI) | **Anticipated absolute effects** | |
|  |  |  |  |  |  |  | **With Ketamine** | **With Midazolam** |  | **Risk with Ketamine** | **Risk difference with Midazolam (95% CI)** |
| **Number of patients with satisfactory separation from parents (CRITICAL OUTCOME)** | | | | | | | | | | | |
| 244 (4 studies) | no serious risk of bias | very serious^1^ | no serious indirectness | serious^2^ | undetected | **⊕OOO** **VERY LOW** | 72/117  (61.5%) | 69/127  (54.3%) | **RR 0.92**  (0.64 to 1.33) | **Study population** | |
|  |  |  |  |  |  |  |  |  |  | **615 per 1000** | **49 fewer per 1000** (from 222 fewer to 203 more) |
|  |  |  |  |  |  |  |  |  |  | **Moderate** | |
|  |  |  |  |  |  |  |  |  |  | **631 per 1000** | **50 fewer per 1000** (from 227 fewer to 208 more) |
| **Satisfactory induction or mask acceptance (CRITICAL OUTCOME)** | | | | | | | | | | | |
| 340 (5 studies) | no serious risk of bias | no serious inconsistency | no serious indirectness | serious^2^ | undetected | **⊕⊕⊕O** **MODERATE**^1^ | 97/165  (58.8%) | 109/175  (62.3%) | **RR 1.09**  (0.94 to 1.27) | **Study population** | |
|  |  |  |  |  |  |  |  |  |  | **588 per 1000** | **53 more per 1000** (from 35 fewer to 159 more) |
|  |  |  |  |  |  |  |  |  |  | **Moderate** | |
|  |  |  |  |  |  |  |  |  |  | **800 per 1000** | **72 more per 1000** (from 48 fewer to 216 more) |
| **Number of patients with satisfactory sedation level (CRITICAL OUTCOME)** | | | | | | | | | | | |
| 481 (7 studies) | serious^3^ | no serious inconsistency | no serious indirectness | serious^2^ | undetected | **⊕⊕OO** **LOW**^1,2^ | 99/243  (40.7%) | 147/238  (61.8%) | **RR 1.53** [1.28 to 1.83] | **Study population** | |
|  |  |  |  |  |  |  |  |  |  | **407 per 1000** | **216 more per 1000** (from 114 more to 338 more) |
|  |  |  |  |  |  |  |  |  |  | **Moderate** | |
|  |  |  |  |  |  |  |  |  |  | **450 per 1000** | **238 more per 1000** (from 126 more to 374 more) |

1 *I*^2^ > 75%
2 Total number of events is less than 300

3 Risk of Bias (High risk)
